# Human Urine Derived Stem Cells in Combination with β-TCP Can Be Applied for Bone Regeneration

**DOI:** 10.1371/journal.pone.0125253

**Published:** 2015-05-13

**Authors:** Junjie Guan, Jieyuan Zhang, Haiyan Li, Zhenzhong Zhu, Shangchun Guo, Xin Niu, Yang Wang, Changqing Zhang

**Affiliations:** 1 Department of Orthopedic Surgery, Shanghai Jiao Tong University Affiliated Sixth People’s Hospital, Shanghai, People’s Republic of China; 2 Med-X Research Institute, School of Biomedical Engineering, Shanghai Jiaotong University, Shanghai, People’s Republic of China; 3 Institute of Microsurgery on Extremities, Shanghai Jiao Tong University Affiliated Sixth People’s Hospital, Shanghai, People’s Republic of China; Instituto de Engenharia Biomédica, University of Porto, PORTUGAL

## Abstract

Bone tissue engineering requires highly proliferative stem cells that are easy to isolate. Human urine stem cells (USCs) are abundant and can be easily harvested without using an invasive procedure. In addition, in our previous studies, USCs have been proved to be able to differentiate into osteoblasts, chondrocytes, and adipocytes. Therefore, USCs may have great potential and advantages to be applied as a cell source for tissue engineering. However, there are no published studies that describe the interactions between USCs and biomaterials and applications of USCs for bone tissue engineering. Therefore, the objective of the present study was to evaluate the interactions between USCs with a typical bone tissue engineering scaffold, beta-Tricalcium Phosphate (β-TCP), and to determine whether the USCs seeded onto β-TCP scaffold can promote bone regeneration in a segmental femoral defect of rats. Primary USCs were isolated from urine and seeded on β-TCP scaffolds. Results showed that USCs remained viable and proliferated within β-TCP. The osteogenic differentiation of USCs within the scaffolds was demonstrated by increased alkaline phosphatase activity and calcium content. Furthermore, β-TCP with adherent USCs (USCs/β-TCP) were implanted in a 6-mm critical size femoral defect of rats for 12 weeks. Bone regeneration was determined using X-ray, micro-CT, and histologic analyses. Results further demonstrated that USCs in the scaffolds could enhance new bone formation, which spanned bone defects in 5 out of 11 rats while β-TCP scaffold alone induced modest bone formation. The current study indicated that the USCs can be used as a cell source for bone tissue engineering as they are compatible with bone tissue engineering scaffolds and can stimulate the regeneration of bone in a critical size bone defect.

## Introduction

Cell-based tissue engineering is a promising alternative approach to facilitate bone regeneration [1–5]. Bone tissue engineering requires the dynamic integration of osteoprogenitor cells and a biodegradable scaffold [6, 7]. Different stem cell types are used in animal models to produce a biomechanical bone construct. Bone marrow mesenchymal stem cells (BMSCs) are widely used for bone tissue engineering [3, 4, 8, 9]. Nevertheless, their sources are limited as they must be obtained by aspirating bone marrow. Embryonic stem cells, which proliferate indefinitely, can be induced to undergo osteogenic differentiation [10, 11]. The application of embryonic stem cells is, however, encumbered by ethical controversy and the risk of teratoma formation [12]. Induced pluripotent stem cells (iPS) were first reported in 2006 and have been demonstrated to be able to differentiate into osteogenic cells [13, 14]. However, using iPS cells for personalized medicine will be exorbitantly expensive, and safety concerns must be resolved before iPS cells for potential clinical use [15, 16]. Therefore, the search continues to identify a readily accessible and abundant source of stem cells to fully exploit the potential of bone tissue engineering.

Recent advances show that urine provides an efficient and convenient source of stem cells called urine-derived stem cells (USCs) as they are available in large numbers and are easily to be harvested [17]. Bharadwaj et al. have demonstrated that USCs possessed self-renewing capacity and multilineage differentiation potential [18]. USCs express urothelial markers after they are seeded onto a bacterial cellulose polymer [19]. In addition, USCs with a small intestinal submucosa scaffold could form a multilayer structure similar to that of native urinary tract tissue [20]. This is a direct evidence to show that USCs can be used as seed cells for urinary tract tissue. Our previous studies have demonstrated that USCs share similar characteristics with adipose derived stem cell (ASCs), which express typical surface antigens of MSCs [21]. Besides, USCs even have a higher proliferation capacity than ASCs. We further found that, under optimal induction conditions, USCs can differentiate into osteoblasts, chondrocytes, and adipocytes. All of these results suggested that USCs represent a promising cell source for cytotherapy and regenerative medicine, including bone tissue engineering. However, the interactions between USCs and biomaterials as well as the potential ability of USCs as seed cells to induce bone regeneration for bone tissue engineering, have rarely been reported.

To select a biomaterial model to evaluate the USCs as seed cells for bone tissue engineering, β-TCP is a good candidate. β-TCP scaffolds have been widely used to repair bone defects in clinical applications with or without cells due to its porous structure and good biocompatibilty [22–25]. Therefore, in this study, we seeded USCs onto a commercialized β-TCP scaffold and investigated the feasibilities to apply USCs as seed cells for bone tissue engineering. We first studied the ability of USCs to adhere, survive, and proliferate on β-TCP scaffolds *in vitro*. We then determined whether USCs seeded in β-TCP scaffolds were capable of differentiating into osteogenic lineage after being cultured in osteogenic media for certain periods. Furthermore, we determined the ability of USCs combined with β-TCP scaffolds to promote bone healing after USCs/β-TCP scaffolds were implanted into rat segmental femoral defects for different times.

## Materials and Methods

### USCs isolation

The Ethics Committee of Shanghai Sixth People’s Hospital, which affiliates to Jiaotong University, approved the use of human urine. Sterile urine samples were obtained from eight healthy donors (5 male and 3 female). Written informed consent was obtained from all study participants. 200 ml urine sample was centrifuged and washed with 40 ml phosphate-buffered saline (PBS). The obtained cell pellets were resuspended and plated in 24-well plates with mixed culture media, which contain Dulbecco’s modified Eagle’s medium (DMEM) culture media supplemented with 2% (vol/vol) fetal bovine serum (FBS) (Gibco, Invitrogen), 10ng/ml human epidermal growth factor (hEGF, Peprotech), 2 ng/ml platelet-derived growth factor (PDGF, Millipore), 1 ng/ml transforming growth factor-β (TGF-β, Peprotech), 2 ng/ml basic fibroblast growth factor (bFGF, Sigma-aldrich), 0.5 μM cortisol (Sigma-aldrich), 25 μg/ml insulin (Humulin), 20 μg/ml transferrin, 549 ng/ml adrenaline, 50ng/ml triiodothyronine, L-glutamine and antibiotics. 5–7 days later, the non-adherent cells were washed out using PBS. Culture media were changed every 3 days. After reaching subconfluence, the cells were passaged using trypsin.

### Analysis of cell-surface antigens

USCs at passage 5 were prepared as described previously [21]. Cells were stained with fluorochrome-conjugated monoclonal antibodies against CD29, CD34, CD44, CD45, CD73, CD90, CD133, and HLA-DR (all from BD) according to the manufacturer’s instructions. After the cells were incubated with antibodies for 30 min at ambient temperature, they were washed with 1% bovine serum albumin (BSA). The cells were then centrifuged and, after discarding the supernatant, resuspended in 1% BSA. Flow cytometric analysis was performed using a Guava Technologies flow cytometer (Guava, easyCyte HT), and the results were analyzed using Cytosofe (Version 5.2, Guava Technologies).

### Induction of differentiation

The potential of USCs to differentiate into osteogenic, chondrogenic, and adipogenic cells was analyzed as previously described [26]. Briefly, to induce osteogenic differentiation, cells were cultured with osteogenic differentiation media for 3 weeks (Life Technologies, Gibco). Extracellular accumulation of calcium was examined by Alizarin Red and von Kossa staining. For chondrogenesis induction, cells were pelleted and cultured in the chondrogenic differentiation media (Life Technologies, Gibco) for 4 weeks. Positive induction was detected by Toluidine blue staining after differentiation. For adipogenic induction, passage 5 USCs were seeded at a density of 5,000 cells per centimeter square and cultured with adipogenic differentiation media for 2 weeks (Life Technologies, Gibco). The formation of lipid vacuoles was detected by Oil Red O staining.

### Cell seeding

Porous β-TCP was purchased from Bio-Lu Biomaterials Company (Shanghai Bio-Lu Biomaterials Co., Ltd., Shanghai, China). The solid porous components of the microstructure were completely interconnected. The pore was 100–400 μm in diameter, and the average porosity was 75%. The compression strength was larger than 2 mPa.

For cell seeding, passage 5 USCs were trypsinized, counted using a hemocytometer, and 5×10^5^ USCs were directly seeded onto β-TCP scaffolds. Cells were allowed to adhere for 2 h at 37°C with 5% CO_2_ incubator before the addition of culture media. For in vitro experiments, USCs/β-TCP composites were either cultured in USCs media as descripted before or incubated with osteogenic media (Life Gibco, Osteogenic Differentiation Kit). For in vivo experiments, the compositions were pre-cultured in the osteogenic differentiation media for 7 days (Life Gibco, Osteogenic Differentiation Kit).

### Visualization of USCs on the surface of β-TCP

Scanning electron microscopy (SEM) was performed to analyze the adherence of USCs. The scaffolds were fixed in 4% formaldehyde for 2 h, dehydrated in a series of ethanol concentrations, lyophilized at -80°C for 24 hours and vacuum-dried overnight. Specimens were subsequently sputtered with gold. The specimens were analyzed using a scanning electron microscope (FEI Quanta 200 FEG).

### Analysis of cell viability

Cell viability assays were conducted using the Live/Dead assay kit (Molecular Probes, Invitrogen) according to the manufacturer’s instructions. Briefly, the composites were washed 3 times with PBS, incubated in 4 μM Calcein AM and 4 μM Ethidium homodimer-1 for 45 min, washed twice with PBS, and analyzed using a confocal microscope (Carl Zeiss LSM 5 PASCAL). In this assay, green and red fluorescence indicates living and dead cells, respectively.

### Analysis of cell proliferation

Cell proliferation was evaluated using a Cell Counting Kit-8 (CCK-8, Dojindo, Japan). After composites were cultured for 1, 2, 3, 4, 5, 6, and 7 days, CCK-8 solution was added to the culture system and the mixture was then incubated at 37°C for 3 h. The optical density (OD) at 450 nm was measured using a microplate reader (Bio-rad, iMark) to evaluate the number of viable cells in the scaffolds.

### Analysis of alkaline phosphatase (ALP) activity and calcium content

The cell/scaffold composites were cultured in control or osteogenic media (Life Gibco, Osteogenic Differentiation Kit). On days 7 and 14, the ALP activity of the composites were determined according to the manufacturer’s instructions (Jiancheng, China) and normalized to total protein content, which was detected using the Pierce BCA Protein Assay Kit (Pierce Biotechnology, USA). Briefly, the composites were washed three times with PBS, smashed into small pieces, and submerged in 0.1% Triton X-100. The solution was transferred into a 1.5 ml tube and centrifuged. The ALP activity and protein concentration of the supernatant were determined using the ALP assay and Pierce BCA kit, respectively. ALP activity is expressed as the absorbance at 450 nm (OD value)/ μg total cellular proteins. All experiments were performed in triplicate.

To further detect the mineralization of USCs, the amount of calcium contents in the scaffolds was measured as described previously [27]. Briefly, the scaffolds were washed thoroughly with PBS. Then the scaffolds were homogenized with 0.6 N HCl and shaked for 4 h at 4°C.The lysate was centrifuged and the supernatant was obtained to measure the calcium content. The calcium concentration was determined by colorimetric assay with cresolphthalein complexone (Sigma). The absorbance was measured at 575 nm using a microplate reader (iMark, Bio-Rad).

### Surgical procedure

The use of laboratory animals was approved by the Ethics Committee of Shanghai Jiao Tong University Affiliated Sixth People’s Hospital. Adult male SD rats (n = 27; weight, 350–450 g) were provided by the Experimental Animal Center of the Shanghai Sixth People’s Hospital. The rats were housed in a temperature and light-controlled environment that was ventilated with filtered air.

Rats were anesthetized by intraperitoneal injection of chloral hydrate. Using a sterile technique, the mid-shaft of the right femur was exposed through a lateral longitudinal skin incision. The bone was stabilized with a stainless plate, which was fixed with screws on the proximal and distal ends of the femoral. After the periosteum was removed, a 6-mm critically sized femoral defect was osteotomized using an oscillating saw as described previously [28]. The rats were randomly divided into 3 groups to receive the following implants: (1) USCs/β-TCP (n = 11), the bone defect was filled with USCs and β-TCP; (2) β-TCP (n = 11), the defect was filled with β-TCP alone; (3) control (n = 5), the defect was untreated to validate the segmental bone defect model. The wound was irrigated with sterile physiological saline solution and the deep muscle layer and skin was separately closed. Animals resumed normal ambulation and behavior within 3 days.

To avoid potential immune rejection of xenogenic USCs, cyclosporin A (10mg/Kg of body weight; Novartis, Switzerland) was administered 3 days before the transplantation and continued until the end of the study. No signs of infections were identified during the course of the experiment. 12 weeks after surgery, all the animals were sacrificed using an overdose of chloral hydrate. Micro-CT analyses were performed after removing the plate and soft tissue surrounding the femur.

### Radiographic analysis and micro-CT measurement

Healing of the femoral defects was assessed using sequential X-ray analysis on weeks 4 and 8 after surgery. X-ray was taken at the same condition (46 kV, 2 mA and 2.79 ms). The X-rays were judged independently by three experienced orthopedic surgeons who were uninformed of the nature of the samples.

### Micro-CT analysis of bone formation

Quantitative bone formation was examined using micro-CT (Skyscan, 1076 scanner, Kontich, Belgium) of femurs12 weeks after surgery. The plate and screw were removed before analysis. Samples were scanned through a rotation angle of 180° using step of 0.6°. The 3-dimensional image was reconstructed using NRecon software (Version 1.5.1.4, Skyscan). A cylindrical region of interest (ROI) was selected for analysis based on the reconstruction data. This ROI was situated at the location of the original defect. The parameters of new bone volume relative to tissue volume (BV/TV) and bone mineral densities (BMD) were used for quantitative analysis of the specimens.

### Histological analyses

The rats were killed 12 weeks after surgery, and their femurs were harvested. The plates and screws were separated and removed. All specimens were fixed in 10% paraformaldehyde. For processing undecalcified resin sections, samples were dehydrated in graded series of alcohol and embedded in methylmethacrylate. Sections longitudinally to the shaft were obtained with a Leica 1600 diamond saw microtome. The sections were then stained with Masson’s trichrome to identify new bone formation. For processing decalcified paraffin sections, femoral bone was fixed in 10% formalin for 48 h and decalcified in 15% EDTA for 3 weeks at 37°C. After serial dehydration, the femoral bones were embedded in paraffin, and longitudinal sections were stained with hematoxylin and eosin (HE).

### Sequential fluorescence labeling

A sequential polychrome fluorescence labeling method was performed to determine the rate of new bone formation and mineralization as reported previously [29]. At 4 and 6 weeks after surgery, the animals were injected intraperitoneally with calcein (25mg/Kg; Sigma-Aldrich) and Alizarin red (20mg/Kg; Sigma-Aldrich). After the samples were harvested, undecalcified resin sections were evaluated for fluorescence labeling using a confocal laser scanning microscope (Carl Zeiss LSM 5 PASCAL).

### Immunohistochemistry

To identify grafted USCs, a mouse anti-human nuclear monoclonal antibody (MAB1281) was used. Briefly, deparaffinized sections were washed with PBS, treated with antigen retrieval, and blocked with mouse IgG for 30 min. Subsequently mouse anti-human nuclear antibody (MAB1281) at a 1:200 dilution were incubated at 4°C overnight. Then the biotinylated secondary antibody and ABC complex were applied and DAB substrate was used to stain the section. The sections were stained with hematoxylin and the results were analyzed with a light microscope.

### Statistical analysis

Values are reported as the mean and standard deviation, and the differences among the groups were evaluated using one-way analysis of variance (SPSS, Chicago, IL, USA). *P* ≤ 0.05 was defined as indicating statistically significant differences.

## Results

### Characteristics of USCs

**Fig 1:**
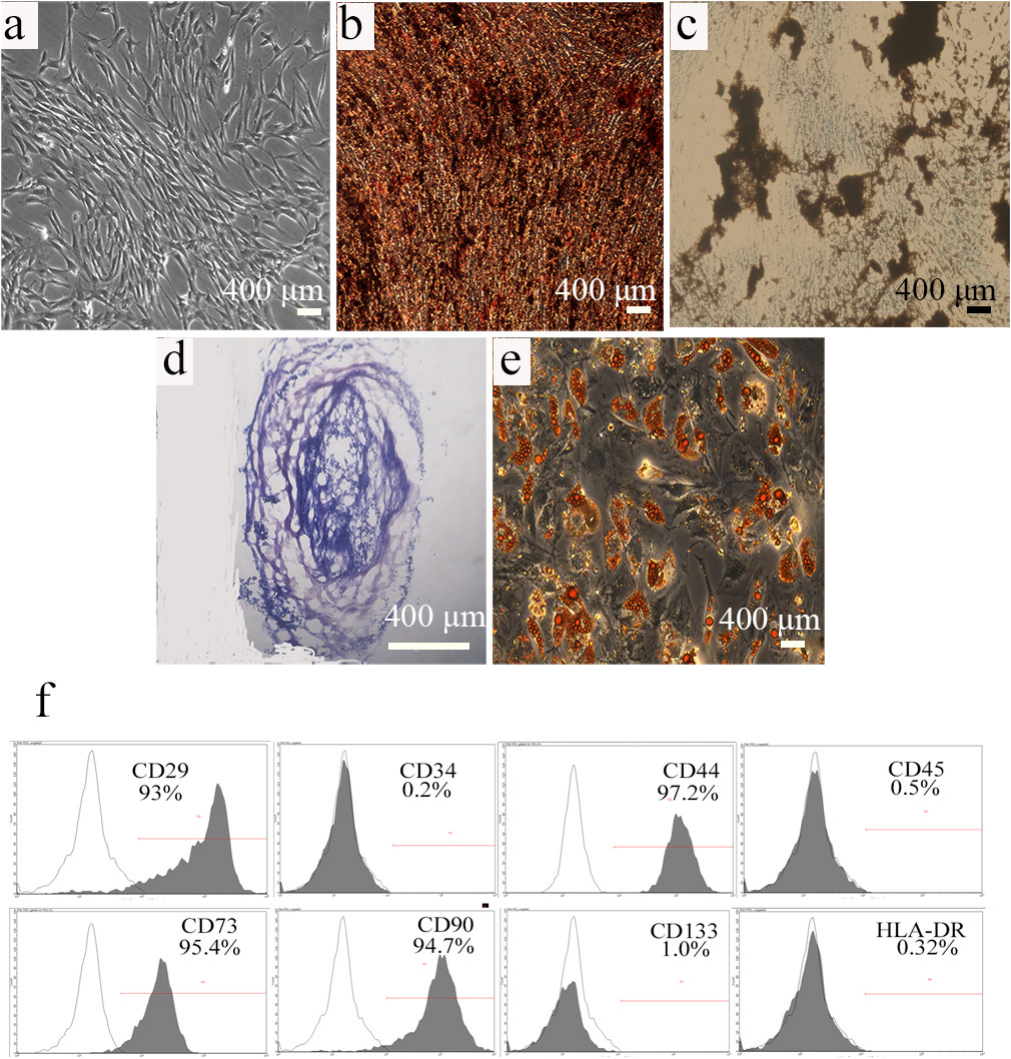
Biological characteristics of USCs. (a) Primary USCs exhibited a spindle-shaped morphology. Osteogenic differentiation was confirmed using Alizarin Red (b) and von Kossa (c) staining, (d) Toluidine blue staining revealed chondrogenic differentiation, and (e) adipogenic differentiation was verified using Oil red O staining. (f) Flow cytometric analysis demonstrates that USCs expressed markers associated with mesenchymal stem cells and did not express HLA-DR. Black traces indicate isotype controls and gray traces show the level of cell-surface expression. Scale bar = 400 μm.

Typical USCs colony-forming units were detected 7–10 days after culture. Observation obtained using an inverted phase-contrast light microscope showed that the morphology of freshly isolated colonies was fibroblast-like (Fig 1A). The USCs were positive for CD29, CD44, CD73 and CD90 and negative for CD34, CD45, CD133, and HLA-DR. (Fig 1F). These finding indicate that the phenotype of USCs was similar to that of MSCs.

To determine the potential of USCs to differentiate into multiple lineages, they were cultured in the appropriate media to induce into osteoblasts, chondrocytes, and adipocytes, respectively. Alizarin Red and von Kossa staining demonstrated the formation of extracellular calcium crystals, indicating osteogenic differentiation (Fig 1B and 1C). Toluidine blue staining detected the presence of glycosaminoglycans, indicating chondrogenic differentiation (Fig 1D). Oil Red O staining demonstrated the presence of lipid vacuoles, indicating adipogenic differentiation (Fig 1E).

### Adhesion, survival, and proliferation of USCs on β-TCP

**Fig 2:**
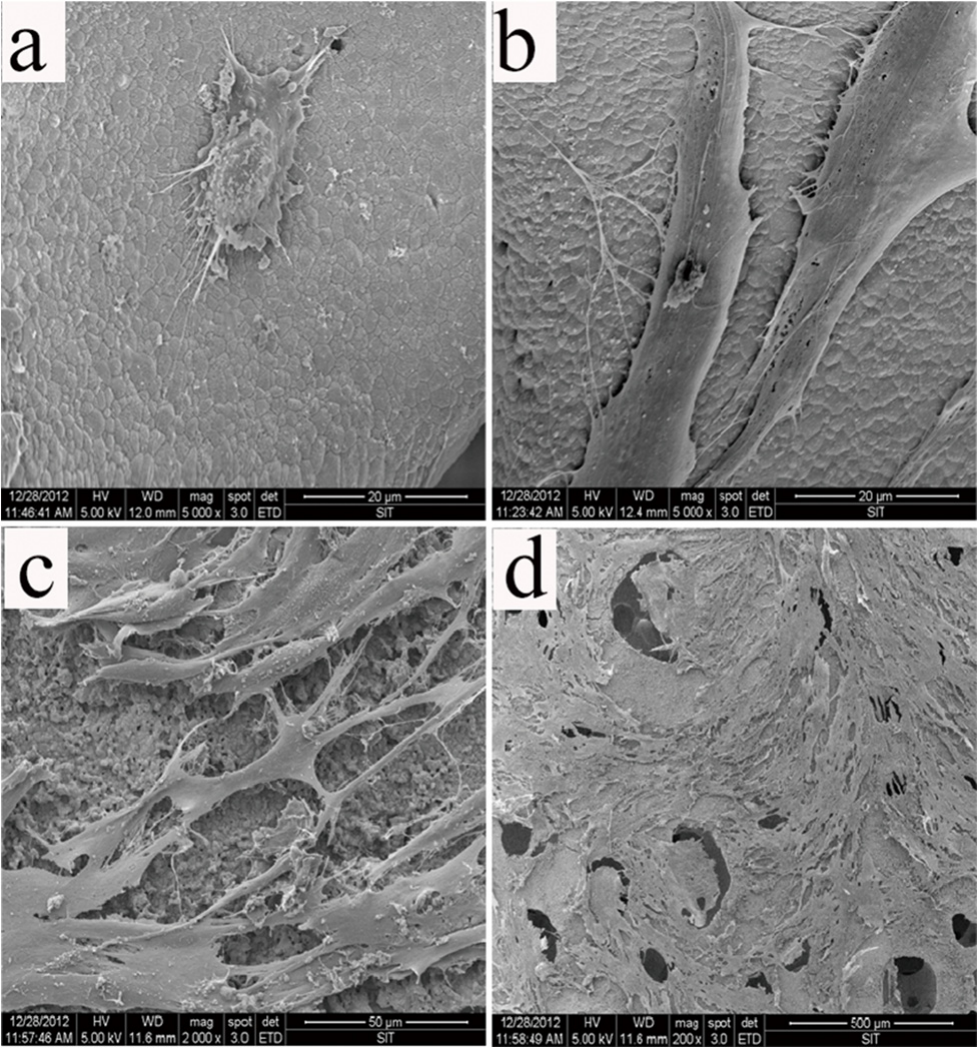
Scanning electron micrographs of USCs on β-TCP scaffold after different incubation time. (a) 12 h, (b) 24 h, (c) 3 days, and (d) 5 days.

**Fig 3:**
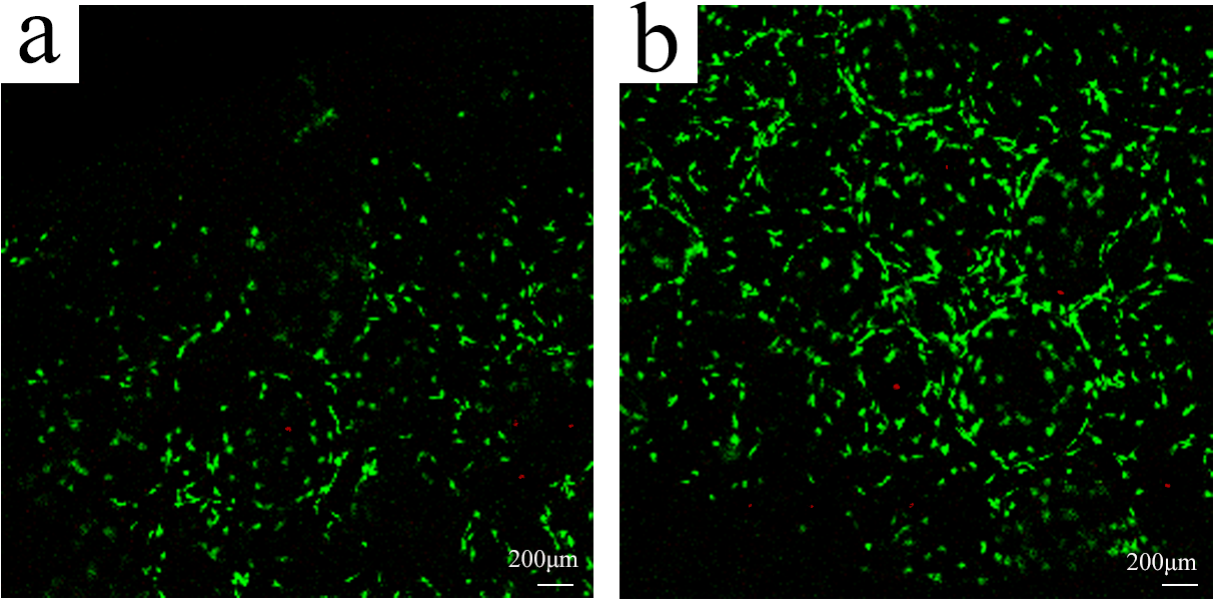
Live/Dead assay for USCs on β-TCP scaffold. (a) 1 day; (b) 7 days. Scale bar = 100 μm.

**Fig 4:**
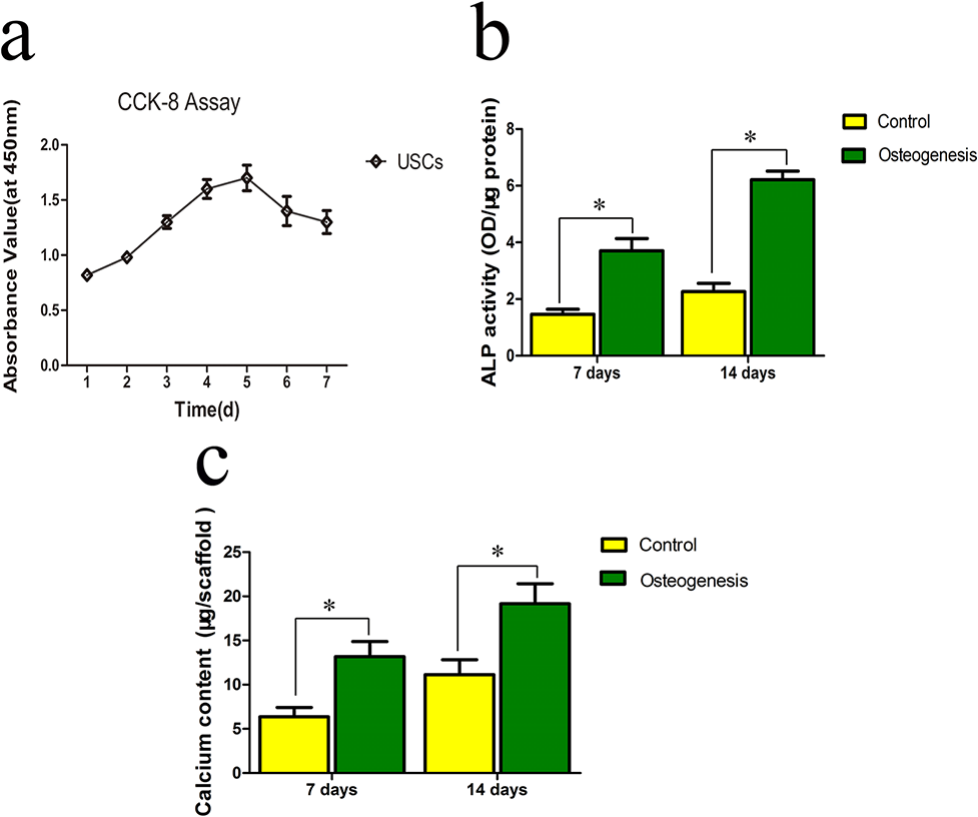
Proliferation of USCs on a β-TCP was determined using the CCK-8 assay on days 1, 2, 3, 4, 5, 6, and 7 (a). (b) Comparison of ALP activity after culturing USCs in osteogenesis media. (c) Calcium content quantification of USCs cultured on β-TCP for 7 or 14 days (*P<0.05).

SEM was used to evaluate the adhesion of USCs on the β-TCP scaffold. USCs extended pseudopodia and adhered to the inner pore wall 12 h after seeding β-TCP (Fig 2A). The morphology of the USCs resembled that of fibroblasts after 24 h (Fig 2B). On day 3, the USCs reached 70–80% confluence (Fig 2C), and on day 5, most of the pores were covered with clusters of cells (Fig 2D). The viability of the USCs was assessed using the Live/Dead Double staining assay 24 h after the cells were seeded on the scaffolds (Fig 3A). The majority of cells retained their viability on β-TCP after 7 days seeding (Fig 3B). The proliferation of USCs on β-TCP was monitored for 7 days using a CCK-8 kit (Fig 4A). The OD value, which reflects metabolic activity and is directly proportional to the number of living cells, increased from 0.8 to 1.7 and then decreased to 1.3.

### Osteogenic differentiation of USCs on β-TCP

Our previous study shows that USCs differentiate into osteoblast in a 2D culture system. To determine whether USCs cultured on β-TCP differentiated into osteoblast-like cells, ALP activity and calcium content were measured. ALP activity increased significantly when scaffolds were cultured in osteogenic media at both 7 and 14 days (Fig 4B). In agreement with ALP activity, calcium content quantification showed substantial levels of calcium deposition after 7 and 14 days of culture in osteogenic media (Fig 4C). This data suggested that USCs on the β-TCP scaffold differentiated into osteoblasts.

### X-ray and micro-CT analysis

**Fig 5:**
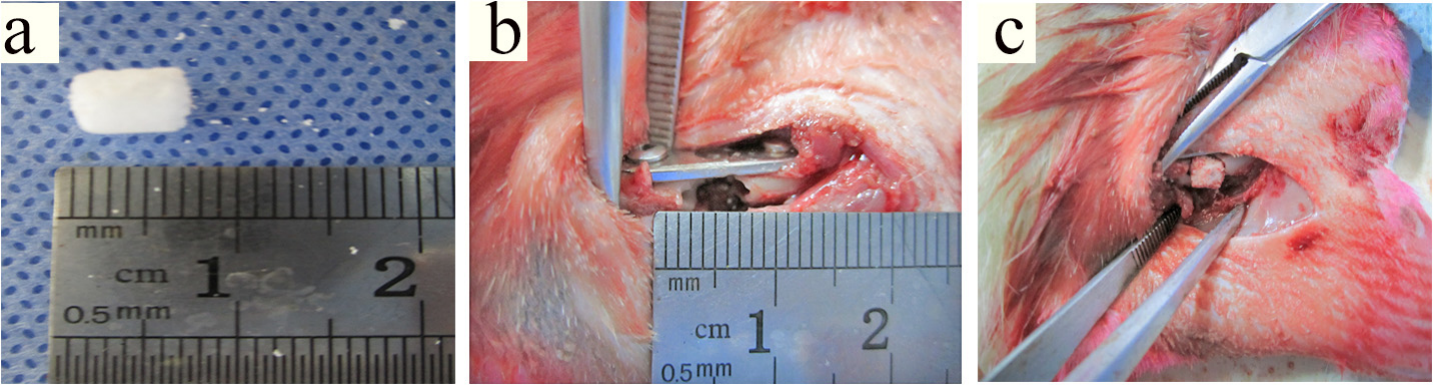
Surgical procedures and in vivo experiment. (a) β-TCP scaffold; (b) femoral defect with a length of 6 mm was created; (c) the bone defect was implanted with USCs/β-TCP, β-TCP or nothing.

**Fig 6:**
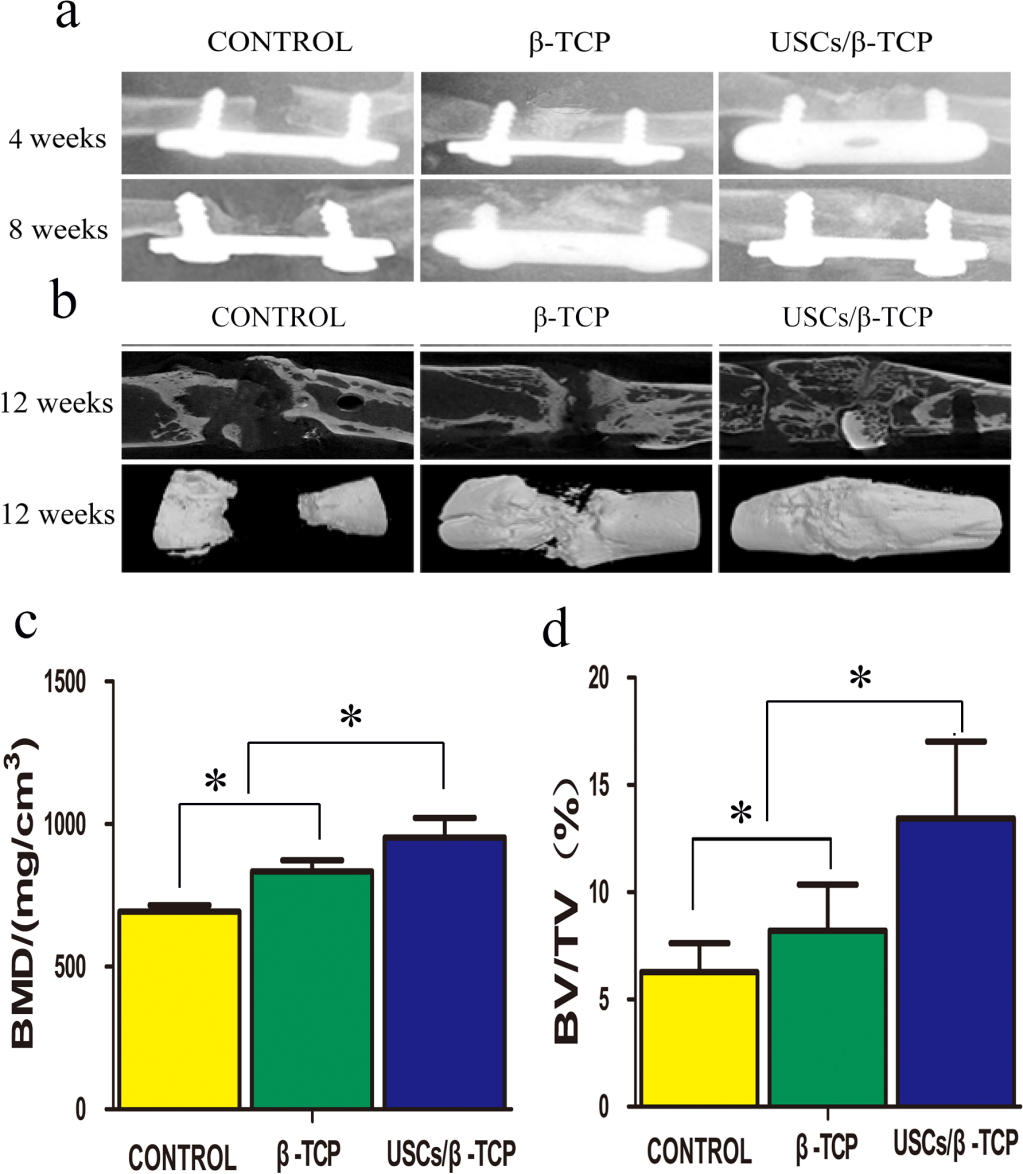
X-ray and micro-CT analysis of femoral defects. The groups are listed on the upper side of image. The times are listed on the right side of the image. (a): X-ray images of the femoral defect shows callus formation and bridging the defect in USCs/β-TCP group. (b): Representative micro-CT slice and reconstructed image show the continuous callus that bridged the femoral defect in USCs/β-TCP group; scaffold only group exhibited a reparative callus but did not show bridging of the defect; and blank control group showed limited callus formation. (c) Bone mineral density (BMD) and (d) bone volume were quantified within a standard ROI placed concentrically over the defect site (*P<0.05).

5 × 5 × 6 mm β-TCP were molded (Fig 5A). Rats 6 mm femoral defects were established (Fig 5B). The defects were implanted with USCs and β-TCP (Fig 5C). The healing process was examined using X-ray analysis at 4 and 8 weeks after implantation (Fig 6A). USCs adhered to β-TCP significantly promoted new bone formation. Bone union was present in 5 of 11 (45.5%) rats (Group USCs/β-TCP), but only 2 of 11 (18.2%) in the β-TCP only group (Group β-TCP).

The harvested femurs were further analyzed using micro-CT to evaluate the amount of new bone formation in the defect site (Fig 6B). Continuous callus formation was detected between the two defect sites, and bony-union was achieved in 5 rats in Group USCs/β-TCP. The β-TCP scaffold was still identifiable after 12 weeks and was incorporated within the new bone. Moreover, new bone was present in the defect site in Group β-TCP only, although the defect site was not spanned. The new bone volume of control group was least. The results of micro-CT analysis at 12 weeks are as follows (Fig 6C and 6D): Bone mineral density (BMD, mg/cm^3^) values for USCs/β-TCP, β-TCP, and empty control were 953.0 ± 120.1, 834.5 ± 67.7, and 693.1 ± 38.6, respectively. The bone volume fraction (BV/TV) was as follows: 16.3 ± 4.2%, 8.2 ± 3.7%, and 6.2 ± 2.4% for USCs/β-TCP, β-TCP, and empty control, respectively. These results reveal that USCs/β-TCP induced osteogenesis to a greater extent than β-TCP alone or empty control.

### Histological analysis

**Fig 7:**
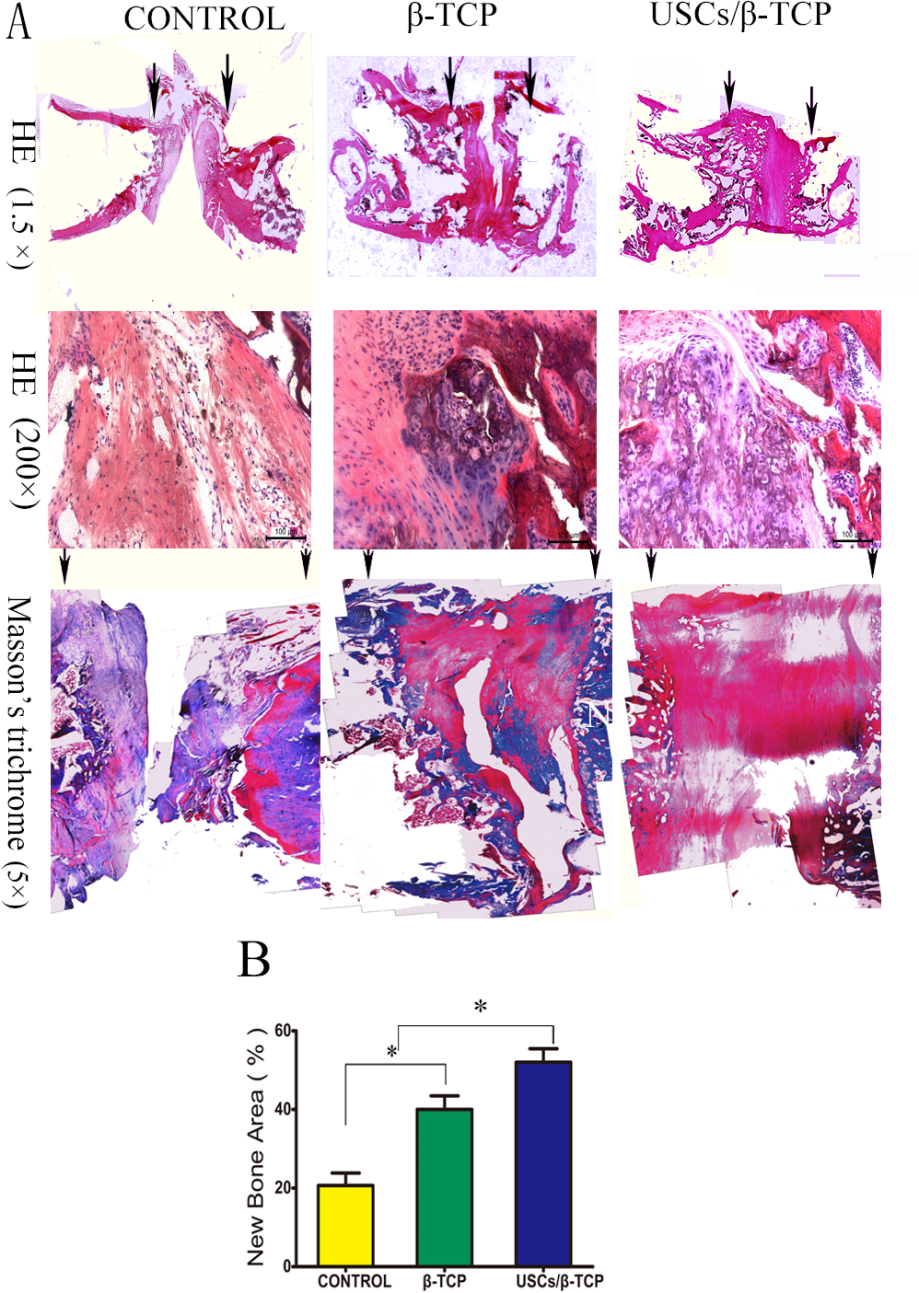
Histological analysis reveals bone formation within empty control, β-TCP and USCs/β-TCP at 12 weeks. (a) HE and Masson’s trichrome staining showing the repair of bone formation in the critical size femoral defect model. Arrows indicate the edges of host bone. (b) The percentage of the new bone area was calculated from the image of Masson’s Trichrome sections (*P<0.05). Scale bar = 100 μm (HE 200×).

12 weeks after implantation, decalcified paraffin sections were stained with HE and undecalcified resin sections were stained with Masson’s Trichrome to identify new bone formation within the defect site. There was no obvious inflammation in either USCs/β-TCP or β-TCP group. A large amount of new bone formation was observed in USCs/β-TCP group (Fig 7A). In β-TCP group, we also detected the regenerated bone but the size was much smaller than USCs/β-TCP group. There was fibrous tissue in empty control group. The quantitative analysis of the bone formation was measured in Masson’s Trichrome staining, which confirmed the results of X-ray and micro-CT image (Fig 7B).

### Mineral apposition rate

**Fig 8:**
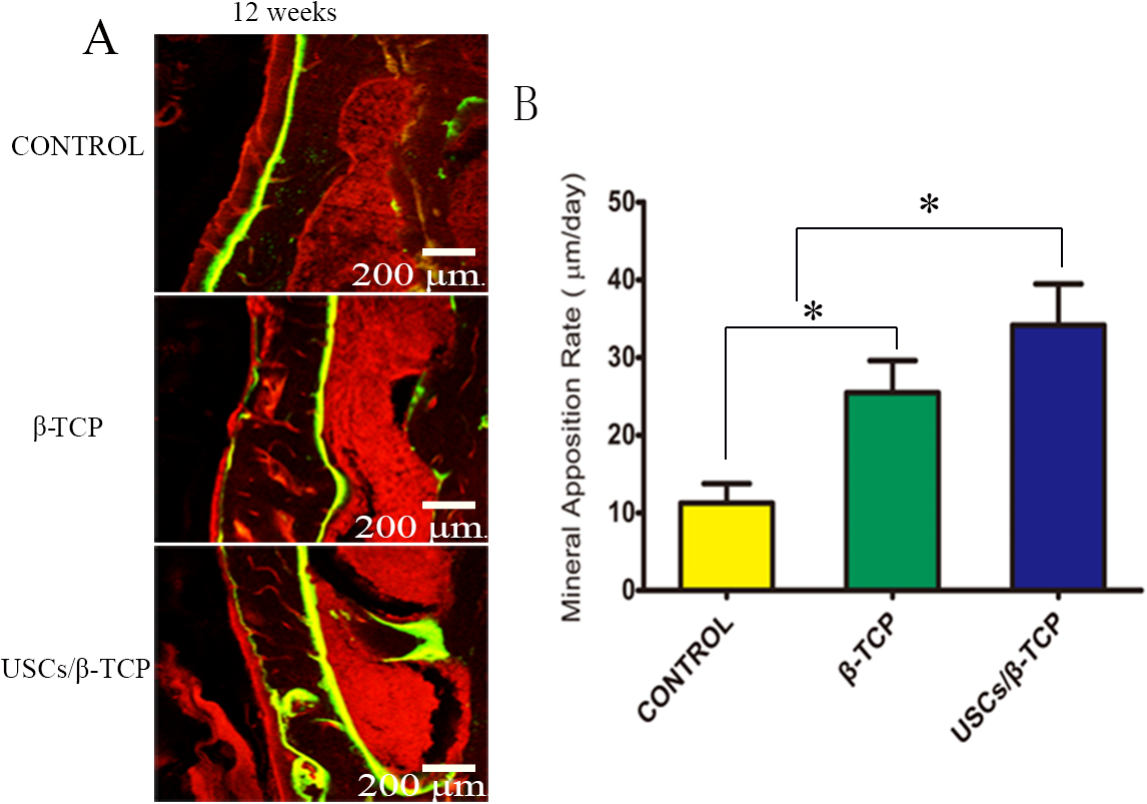
Mineral apposition rate. (a) Alizarin red and calcein reveal mineralization. (b) Quantitative analysis shows a significant difference in the appositional rate of empty control, β-TCP and USCs/β-TCP (*P<0.05). Scale bar = 200 μm.

Fluorescence microscopy detected the calcein and Alizarin Red of all groups, indicating the presence of mineralization and bone formation (Fig 8A). Moreover, the mineral apposition rate for control, β-TCP, and USCs/β-TCP was 11.2 ± 4.3, 25.4 ± 7.2, and 34.2 ± 9.2 μm/day, respectively. The rate of USCs/β-TCP group was significant higher than that of β-TCP or empty control group (Fig 8B).

### In vivo detection of USCs

**Fig 9:**
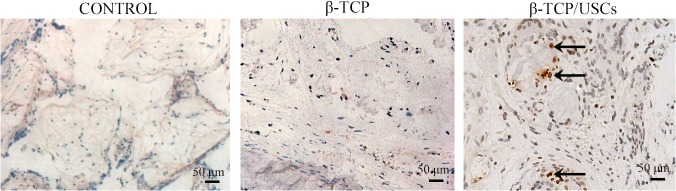
Immunohistochemical staining with MAB1281 at different group after 12 weeks. USCs were identified by arrows. Scale bar = 50 μm.

A human cell nuclei marker (MAB1281) was used to detect the USCs in vivo. Rats treated with blank control or β-TCP did not show any MAB1281 (Fig 9). In contrast, human cell nuclei marker-expressing cells were observed in the defect site of USCs/β-TCP group, indicating the survival of USCs in the defect site.

## Discussion

In the present study, we demonstrated that USCs could serve as potential cell source for bone tissue. We show here that USCs adhered, survived, and proliferated on β-TCP. USCs that were induced to osteoblast with biodegradable β-TCP were able to repair critical size bone defect. In contrast, β-TCP alone was not sufficient to bridge the segmental bone defect. To the best of our knowledge, this is the first report that demonstrates the use of USCs as seed cells for bone tissue engineering and the application of USCs/β-TCP composite to repair a critical size bone defect.

Tissue-engineered bone may replace autografts and allografts for reconstructing bone defects. However, translating tissue engineering from bench to bedside is an expensive and challenging task that is hampered by the lack of a suitable osteoprogenitor cell. Currently, the most frequently used osteoprogenitor cell is harvested from the iliac crest, which requires painful and invasive surgery. The present study indicates that such procedures can be avoided as USCs provide an alternative cell source for bone tissue engineering. Moreover, USCs can differentiate into osteoblast in β-TCP scaffold. Further, we demonstrate here their potential suitability for bone tissue engineering in human, because they induced substantial new bone formation and bridged the critical size bone defect in a rat model system. Compared with other stem cells, USCs can be conveniently and safely isolated using a noninvasive and economical procedure. Further, USCs proliferate exuberantly and can be induced to differentiate into multiple lineages. Therefore, USCs represent an attractive source of cell source for bone tissue engineering.

The success of bone regeneration requires sufficient numbers of osteoprogenitor cells and an appropriate supporting scaffold. Clinical experience demonstrates that autografts, which preserve osteoprogenitor cells, help achieve better repair results than devitalized allografts [30]. For example, investigations of the effects of autologous BMSCs on the healing of critical size bone defect in adult dogs show that, after 4 months, bony union is established in dogs implanted with scaffolds seeded with cells. In contrast, bone union never occurs in dogs implanted with scaffold alone [31]. Further, when adipose stem cells are seeded onto a scaffold and transplanted into a calvarial defect site in rats, more callus forms in rats treated with osteoblasts than in those treated with scaffold alone [4]. These findings are explained by the requirement of large number of osteoprogenitor cells for bone healing, which transform into osteoblast and secrete mineralized matrix [32]. Osteogenic cells migrate into the scaffold to repair a small defect; however, in a segmental bone defect, the scaffold degrades faster than new bone formation and fibrous instead of bony union occurs.

There is controversy regarding whether undifferentiated or osteogenically stem cells are more suitable for bone regeneration. When cultured in osteogenic media, progenitor cells differentiated into the osteogenic lineage. In contrast, undifferentiated cells maintain high proliferation potential. For example, culturing stem cells in osteogenic media yielded the best outcome for regeneration in rats with cranial defect [33]. Studies using transplanted undifferentiated amniotic fluid stem cells (AFSCs) or cells with an osteogenic phenotype to repair a critical size femoral defect in rats show that osteogenically committed AFSCs achieve the best results [34]. To promote the bone healing potency of the transplant cells, we cultured USCs/β-TCP composites in osteogenic differentiation media for 7 days. Further studies are required to investigate which kinds of USCs are better for bone regeneration.

Scaffolds also play a critical role in tissue reconstruction. However, there is debate regarding the ideal scaffold for bone tissue engineering [35, 36]. The structure of β-TCP scaffold is porous and mimics trabecular bone tissue, and its biocompatibility facilitates cell adherence, survival, and differentiation, all of which ultimately promote bone regeneration [23]. Our results show that USCs proliferated robust on β-TCP scaffold. It remains to be determined whether better scaffolds exist for propagating and differentiating USCs. However, β-TCP provides a favorable microenvironment to facilitate USCs growth and differentiation in this study.

To test the ability of USCs/β-TCP combination to induce bone regeneration, we chose critical size femoral defect as our model, because its most common site is long bones, and the repair process for these bones is more complicated than that of flat bone [37]. Here, we show that USCs/β-TCP transplants increased new osseous formation and that 5 out of 11 transplants completely bridged the critical size bone defect. Moreover, USCs/β-TCP was significantly more effective than β-TCP alone. Because bony union did not occur in control group, our model serves as an informative experimental platform for studying the repair of critical size bone defects. In addition, long bone formation occurs mainly through endochondral ossification in which the progenitor cells give rise to cartilaginous structures first, which in turn become ossified and form bone [38]. Cartilage tissue was detected here at the sites of transplantation 12 weeks after rats were transplanted with USCs/β-TCP composite (Fig 7). This finding provides direct evidence that the bone formation occurs through the endochondral ossification process.

In the present study, we transplanted human USCs into Sprague Dawley rats with a femoral defect. Although the immunological properties of USCs are unknown, adult stem cells are immunologically privileged [39]. Moreover, the lack of expression of HLA-DR suggests that USCs are not immunogenic and may be suitable for xenotransplantation. Further, to prevent rejection, we injected cyclosporin A before and after the transplantation. This strategy was based on the findings that the surviving graft cells were present in all of the rats that received brain transplants of human embryonic stem cell-derived neural progenitors were also administered cyclosporine A [40]. Moreover, when rats were administered cyclosporin A before and after they received liver transplants of human MSCs, immune rejection was reduce sufficiently so that human MSCs survived and differentiated into hepatocytes [41]. Further, high doses of cyclosporine inhibit immunocyte infiltration and enable human-rat xenotolerance [42]. In the study, we did not encounter immune rejection, and the outcomes demonstrated that USCs participated in bone regeneration.

Culturing of cells with animal serum is still common for cell research and therapy. It has been demonstrated that the use of animal products has a high risk of virus transmission [43]. Previous USCs culture media composed keratinocyte-serum free media and progenitor cell media, which contained growth factor and fetal bovine serum [17]. In the current study, we used culture media free of fetal bovine serum. The current media avoid the risk of disease transmission. With the development of the enzyme and cell engineering, the quality of growth factors has been greatly improved while the prices have decreased a lot. We can obtain 1 to 2 progenitor cells in 100 ml urine. We estimate that 30 million USCs can be harvested after 4 weeks in vitro expansion. Therefore, USCs can be a promising cell source for bone tissue engineering.

To avoid the influence of other biological substances on USCs, we immediately extracted the cells after obtained the urine and washed several times with PBS. The flow cytometry analysis showed the purity of cells was over 95%. Lang et al reported that up to 75% of fresh USCs can be safely persevered in urine for 24 hours, which indicate these cells stored in urine retain their original stem cell properties [44]. We amplified USCs until passage 5 to get enough cells for the in vitro and in vivo experiment. Meanwhile, USCs in passage 5 still have robust proliferation ability. There is no significant change in surface markers, proliferation ability and differentiation potential of USCs at passage 3 and 5 in our unpublished data. USCs were seeded onto β-TCP scaffold and were cultured in vitro in osteogenic media for 7 days. In vitro results showed that USCs were committed to the osteogenic phenotype after 7 days in culture. Live/dead staining assay indicated that most USCs were still alive. Given those factors, we chose cultured 7 days in vitro and then transplanted the cells and scaffold in vivo.

## Conclusion

The present study concludes that USCs can be used as a seed cell source for bone tissue engineering as they can adhere, proliferate and differentiate into osteoblasts on β-TCP scaffolds. In addition, the combination of USCs with β-TCP could improve the bone regeneration in a critical size defect. Since USCs are very easily to be obtained in a noninvasive and simple procedure, together with their abilities to interact with biomaterials and induce bone regeneration, USCs offer a promising alternative cell source for bone tissue engineering.
